# Supplementary material for: Between patterns and predictions: Interpretable latent EEG representations for clinical insights
Source: Neuroimage Clin. 2026 Oct 14;52:104077. doi: 10.1016/j.nicl.2026.104077 (PMC13694799; doi:10.1016/j.nicl.2026.104077)
Supplement: Supplementary file 1 [file mmc1.docx]

**Supplementary Figures**

**Figure S1: Pathological states.** **A** Representative 10-second EEG segments illustrating six pathological patterns: Burst Suppression (BS), Seizure (SZ), Generalised Rhythmic Delta Activity (GRDA), Lateralised Rhythmic Delta Activity (LRDA), Generalised Periodic Discharges (GPD), and Lateralised Periodic Discharges (LPD). Signals were bandpass filtered (0.5–40 Hz).

**Figure S2: Physiological states.** Representative 10-second EEG segments illustrating physiological brain states across the sleep-wake spectrum: Wakefulness (W), NREM Stage 1 (N1), NREM Stage 2 (N2), NREM Stage 3 (N3), and Rapid Eye Movement sleep (REM). Signals were bandpass filtered (0.5–40 Hz).

**Figure S3: Cross-validation and bootstrap sensitivity analysis for outcome discrimination across three cohorts. Each row corresponds to one cohort: cardiac arrest (top), subarachnoid hemorrhage (middle), and routine EEG (bottom). A Bootstrap ROC curve with 50 iterations showing mean AUC and 95% confidence interval. B Same analysis with 100 bootstrap iterations. C Same analysis with 1000 bootstrap iterations. Panels A-C demonstrate that confidence interval estimates are stable across iteration counts, justifying the use of 50 iterations. D 5-fold cross-validation ROC curves using the three pre-selected features reported in the manuscript. The LDA was fitted on four folds and evaluated on the fifth. Individual fold curves are shown alongside the mean. E 5-fold cross-validation with per-fold brute-force feature selection. For each fold, the best three features were identified on the training set and the LDA applied to the held-out fold. F Histogram showing how often each feature was selected across the five folds in panel. The blue triangles mark best performing features for an LDA trained and fitted to all data, as reported in the manuscript. E. Features selected consistently across folds indicate robust discriminative value independent of the specific train/test split.**

**Figure S4: LDA for different number of features. Discrimination performance (AUC) versus number of selected features for three datasets: Routine EEG, SAH and CA. For each feature count (2, 3, 5, 10), the best-performing feature combination was identified via Linear Discriminant Analysis. All three datasets show only marginal improvement beyond three features, confirming that this choice represents a practical optimum. The constraint to three features in the manuscript enables direct visualization in 3D space for clinical interpretation.**
